# Surgical Observations, &c.

**Published:** 1817-04

**Authors:** 


*Surgical Observations, &c.* By Charles Bell. Part II. Octavo, pp. 100, with four Plates.

Mr. Bell proceeds in his reports with spirit; we shall follow him with patience: but the price is really enormous. Societies, students, and a few of the upper class of Surgeons will take in the publication—all the other branches of the profession are excluded by the expence.

This number contains two Reports, the fifth and sixth. The former is on fractures and dislocation of the spine, fractures of the ribs, emphysema, &c. This we shall first analyse.

1. Fracture of the spine is a most dreadful injury, but not entirely a hopeless one.

Case 1. A young man fell from a height of forty feet, and struck his back against the corner of a stone stair, eighteen feet from the ground. When brought to the hospital, a swelling over the lower dorsal vertebræ; a depression between the spinous processes; great pain in the part and over the abdomen; breathing and sensibility natural; motion and feeling of the lower extremities unimpaired. V. S. ad ℥xvj; leeches to the back; cathartic. Next day, (Sept. 13) reports a restless night; great pain; vomiting; bowels not opened: an enema.—14th. Delirious; pulse frequent; skin hot; passes fæces and urine involuntarily; use of the limbs still perfect.—15th. Very ill; pulse 136; delirious during the night—is now in a state of great excitement.—17th. Is dying.

Dissection. Eleventh dorsal vertebra fractured in its body; spinous process crushed; spinal marrow not apparently injured; thick green pus between the theca and spinal marrow; effusion of serum betwixt the membranes of the brain.

Case 2. S. B. a girl, ætat. 18, was thrown from a window two stories high, and fell on her back. Great tumefaction over the lower dorsal vertebra; one of the spinous processes crushed; spine, above and below, remarkably prominent. Lower extremities not paralytic; all her sufferings referred to the back and loins. Twelve leeches to the injured part, and repeated for three successive days. She lay for weeks complaining and groaning in a pitiable state, while nothing could be done but leech, poultice, and foment, keeping the bowels and skin open, and giving an occasional opiate. She lay on her side for three months, her body bent forward and knees drawn up. At the end of eight months, the lower extremities were contracted, back bent, emaciated; two spinous processes project, leaving an interval of two inches; is syphilitic.—Sept. 15. Is dismissed perfectly well. The extremities of the spinous processes have approximated; she walks erect, and has recovered her shape.

Case 3. T. W. admitted Sept. 24th. He fell from a height of two stories, and came with his back upon the pavement. No injury of the spine could be perceived; but sensation and motion were lost in the lower half of the body. Bladder and intestines insensible to their natural stimuli; pain in the back referred to the middle dorsal vertebra. During six days he was repeatedly cupped, the bladder emptied, and stimulating injections thrown up. At the end of this time his breathing became affected, with cough. Expectorants and opium gave no relief, nor did repeated venæsections. He was quite sensible; lower extremities motionless and insensible; belly full; respiration performed by the heaving of the chest. Died on the 8th of October.

Dissection. Much coagulated blood lay over the 6th and 7th dorsal vertebræ; their spinous processes were broken—tube of the spine forced in on the cord, which a sharp portion of bone had pierced; a rib fractured on the left side.

Case 4. A man was brought to the hospital, July 22, quite dead; frothy blood coming apparently from the lungs. Nothing unusual was found in the abdomen, thorax, or head; but a tumour appeared in the posterior mediastinum, resembling an aneurism of the aorta. It contained a thick mass of scrofulous matter, in contact with a carious portion of the spine. The bodies of the vertebræ were found eroded; the inter-vertebral substance destroyed, and the spinal marrow exposed.

Case 5. *Fatal Diastasis of the Spine.* March 29th, M. ——, a coal waggoner, was admitted. History confused. He had been pitched from his cart, and fell on the back of the neck and shoulders. He could not now stand; complained of pain in his loins, but no injury was perceptible there. Swelling and discolouration between the shoulders. Leeches between the shoulders; bowels opened. For a week he lay without complaining of any thing but stiffness in the back of the neck. No paralysis; could move upper and lower extremities; retain and expel urine and fæces.—8th. Instantaneously affected with convulsions all over the body. Relieved partially by bleeding; continued sensible; jaw locked. Pulse very strong. Convulsions returned; was again bled. After the second bleeding the jaw relaxed, and moved with rapidity. He began to speak with great animation, but appeared maniacal. He passed much fæces and flatus, with singular force. In an hour became composed. Next morning he had symptoms of typhoid fever. On the third day from the attack of convulsions, he had difficulty in using his arm; and in two days more, total palsy of the lower extremities. He died in about a week, retaining characters of typhoid fever till the last.

Dissection. Nothing remarkable in the head, except a slight effusion between the pia mater and tunica arachnoides. A loosening of the last cervical from the first dorsal vertebra, whence some puss issued. The intervertebral substance quite destroyed, and much pus surrounding the vertebræ. The pus had pervaded the whole theca to the cauda equina.

Case 6. A man, in trundling a wheelbarrow, fell with his chin upon the curb stone—was instantly motionless, and brought into the hospital dead.

Dissection. The dentiform process of the second vertebra had burst from its position, torn the transverse ligament, and crushed the medulla oblongata.—This case we should hardly have thought worthy of a place in our *Porte-Feuille*.

Case 7. F. —— fell down a shaft full forty feet in depth. Complains of having hurt his back. Uneasiness and defect of motion in the lower extremities. Cathartic; to be cupped. In eight days complained of languor, universal pain, sickness and debility. Pulse quick; skin hot. Experienced an attack of typhus. After this was over, he still complained of pain in his loins and torpor of the lower extremities. Scarifications, cupping, stimulating liniments cured him, and he was discharged. We should have refused this case a place in any part of our Journal, were it not to shew Mr. Bell the danger of *filling up* his six shilling pamphlets with such wretched stuff. The next is equally uninteresting; but, what is worse, it is much longer: and then follows the outline of a case from the third volume of the *Medical Observations and Inquiries!* Surely, the practice of a metropolitan lecturer might afford more instructive memoranda than are here detailed.

*Review.* Here we have some sensible observations; but with a tincture of sarcasm, not to say illiberality. Our author knows of no *books* to which he can refer his pupils for information on this subject.—"Authors have treated of the subject very superficially; and we have only some occasional cases in our books." Now we believe that any periodical Journal, on either side of the Tweed, could furnish more interesting cases of spinal injuries than Mr. Bell has brought forward. His remark, therefore, savours of arrogance; and much as we admire his talents, we shall not hesitate to tell him his faults.

The danger which is to be apprehended in injuries of the spine is the same with that which accompanies injuries of the brain, viz. the inflammation and suppuration within the theca; hence the necessity of general, and still more of local bleeding, while the danger continues. To these must be enjoined absolute rest. Our author does not see much analogy between fracture and depression of the skull, and fracture of the spine with crushing of the spinal marrow, as far as regards an operation. Neither do we. He thinks that the removal of a portion of bone surrounding the marrow would be inevitably fatal, since its membranes are the most susceptible of inflammation of any in the whole frame. Besides, when a portion of skull is depressed, a comparatively small portion of the brain is injured by the intrusion; whereas the whole diameter of the spinal marrow is generally crushed when the canal is at all infringed on. The exposure, therefore, of the spinal marrow would, he thinks, be followed by inflammation, suppuration, and death. The foregoing cases shew the inaccuracy of the diagnoses laid down by authors:—In what disease do we not perceive this inaccuracy? Every Tyro will tell us, that paraplegia and distention of the bladder and intestines result from spinal fracture. Yet, here we see instances of fractured bodies of the vertebræ without any diagnostic symptoms. Instead of loss of motion and feeling, we have seen the patient tossing in restless agony; instead of palsy confining the lower extremities, we find him struggling to get out of bed, and writhing in wild delirium! Dissection shews that the injury done to the spine is followed by inflammation, which is rapidly propagated along the membranes of the spinal marrow, terminating quickly in suppuration.—Hence the general irritation and palsy which ensue. The inflammation of the spinal marrow is attended with an almost universal nervous excitement, which is presently followed by excitement of the brain; this last ending in effusion and death.

*Emphysema from fractured Rib.* CASE 1. A man, 65 years of age, fell from a ladder and struck his left side upon the corner of a chair. In three days he was admitted into the hospital. He now suffered much from pain in his side, aggravated by a dry cough. The ribs could not be felt, but the emphysematous tumour that covered them, evinced a subjacent fracture. The skin was blown up from the ilium to the clavicle. As he could lie down, and was easy, except from the cough, Mr. B. was content with ordering him to be bled and to have a linctus. Next day, the tumour spread further over the breast and hips.—On the 6th and 7th day from the accident, the emphysema began to dissipate, and by common attention to confine the motion of the rib, and keep the circulation low, he quite recovered.

Case 2. July 6th, —— Lynn, a labourer, fell from a scaffolding ten feet high into an area below, and struck his side against an iron bar. He was conveyed to the hospital, bled, and gradually recovered his senses. One or two ribs were fractured—a roller was applied, and he went home.—*On the third day,* it was reported that he was worse, having been delirious all night. He was found labouring under great dyspnœa, with a slight emphysema extending over the right side. Re-application of the roller; opening medicine.—*Fourth day.* Emphysematous crepitation; the tumour extending widely in every direction. His respiration was now very laborious, with a rattling in the throat, and constant expectoration of mucus. Countenance anxious and bloated; pulse 120, hard, and contracted; skin hot and feverish; pain in the right side. V. S. ad ℥xx, with temporary relief. *Ten, p. m.* The patient has once or twice jumped out of bed, quite deranged and apparently suffocating. The blood last drawn inflamed. V. S. ad ℥xviij, with some relief, as before. The bandage could not be borne an instant. He now lies panting and struggling for breath, constantly spitting up frothy and yellow mucus.—*Fifth day.* He fainted last night, but soon recovered, and passed an easier night. This morning he is pale, breathes with difficulty, and lies half comatose.—*Sixth day.* Has passed a bad night; seems sinking; is delirious. Died at eleven o'clock.

Dissection. The whole trunk extremely swollen with emphysema, particularly the right side. On opening the right cavity of the chest, the pleura was accidently punctured, and gave exit to a great quantity of confined air. The right lung was quite collapsed; the left side natural. From the side four ounces of blood were sponged up, supposed to have come from the intercostal artery. A portion of lung was lacerated, and lying in contact with the rugged edges of two fractured ribs. There were five other ribs fractured besides those which penetrated the lungs. Viscera of the abdomen sound. We need not observe that the treatment of this case was not under Mr. Bell. We shall abstain from any remarks on the conduct of the dresser or house-surgeon, who attended this unfortunate patient. Mr. Bell, in his observations on this case, says,—

"1st. The extremity of the broken rib wounds the surface of the lungs. 2d. The enlargement of the walls of the chest in inspiration *sucks the air through the lungs*, and by successive motions accumulates it in the cavity of the chest."

Now, we maintain, that this is a most unphilosophical explanation; and moreover, that it is wrong. It is morally impossible, that the expansion of the chest can suck air from the lung, as long as that lung is unconfined, and capable of following the enlarging dimensions of the cavity in which it is confined. It is during *expiration*, when the lung is compressed on all sides, that air passes partly out of the trachea, and partly out of the wound, accumulating in the bag of the pleura till the lung has no longer space for expansion. This, of course, is attended with a disturbance of the other side of the chest. The mediastinum is pushed on one side, the diaphragm thrust down, and the capacity of that other side, and of the lungs, so much diminished, that the patient is at length suffocated. Here is the triumph of surgery. One stroke of the scalpel, by opening the side of the chest, would have instantly relieved this poor man's sufferings. How the case could have been taken for pulmonic inflammation, God only knows!

Case 3. D. R. ætat. 50, November 18th, fell from a height of ten feet on some iron bars. When brought into the hospital, he was very pale and agitated, complaining of dyspnœa, and pricking pain in the left side. The 7th and 8th ribs were found fractured; a crepitous swelling over the left side of the body. V. S. ad ℥xvj. Tumour to be deeply punctured in three different places.

19th. Tumour not increased; air has escaped in great quantity from the punctures. Pulse calmer; can lie down. He gradually got better, and was discharged on the 4th of December.

Mr. Bell winds up the subject with the following remarks.

1. "In slight degrees of emphysema, the patient generally does well. The practice is as in a common case of fracture of the rib. He is bled again as the pulse rises, or when the breathing is oppressed; the chest is swathed, that he may be forced to breathe with his diaphragm, and give rest to the rib.

2. "That in cases where the air extends far, still it is only frightful, and nothing need be done; but if with this there be much oppression of breathing, we puncture and press out the air in the neighbourhood of the fractured rib.

3. "That if the patient be still oppressed, we relieve him by giving free exit to the air, from the side distended, and consequently permit freer action of the other side of the chest.

4. "That on all occasions, when emphysema appears, the patient requires to be strictly watched; for there may come a rapid increase of difficulty of breathing, and the patient be suddenly cut off."

*Dislocation of the Ribs from their Cartilages.* J. H. ætat. 46, in running across the horse-way of a mill, was caught betwixt the beam and the wall, squeezed into a space of *five inches,* and so wedged as to stop the horse When brought into the hospital, he was pale and breathless; a cold sweat bedewed his face and body; chest felt like a dead body, where the thorax had been opened, and the sternum left loose under the integuments. The ends of the ribs, at their junction with the cartilages, stood out distinctly marked and prominent. The clavicle of the left side was dislocated from the acromion scapulæ. He had cough and pain in breathing. V. S. ad ℥xvj; a roller round the body. Shoulders braced back. He passed a restless night; but next day his breathing was easy, and the pulse more steady. Twelve leeches to the sternum. He gradually recovered.

*Fracture of the Ribs neglected and fatal.* This was the consequence of a boxing match. About three months after the blow, a colourless swelling arose on the right side, attended with pain. In six months the swelling was punctured, and a Scotch pint of matter discharged. The surgeon said the ribs had been fractured. His breathing now became short and oppressed, with much inward pain. At the end of nine months, when Mr. B. first saw him, his state was as follows:—There are several holes on the side of the chest, the mouths of sinuses communicating with each other, and leading to the surfaces of several carious ribs. In three months from this time, the symptoms were those of confirmed hectic from suppuration within the lungs.

Dissection. The third and fourth ribs were fractured, and their surfaces carious to some extent. The bones were black, spongy, and immersed in fœtid matter. The lungs of that side were adherent to the ribs, firm, and consolidated by inflammation; adherent to the pericardium, which was inflamed, thickened, and distended with purulent serum. This lung also contained a large abscess, which was in connexion with the communication betwixt the carious ribs; and it was from this abscess that the copious discharge of matter had flowed out through the wound. The other lung was sound. Mr. Bell says that some were of opinion that there had *not* been a fracture of the ribs originally here; and we join those in opinion, and that from a dissection which we ourselves made a few weeks ago, almost precisely similar to the above, but where no blow or fracture could possibly be suspected. We refer Mr. B. and our readers to *Porte-feuille*, No. 3, "*Abscess of the lungs bursting outwards, and rendering carious the ribs, &c. &c.*" Page 254.

### SIXTH REPORT,

### CONTAINING CASES OF FEMORAL HERNIA.

Mr. Bell justly observes, that the pupil, after making himself acquainted with all the fasciæ and ligaments about the groin, has still to commence the study of hernia, "which has very little to do with the natural anatomy." The great difficulty—the great question in hernia is—when should the operation be performed? To be able to distinguish symptoms, we must attend to their cause, which is in the intestine. The changes in this, our author thinks, have not been inquired into with that diligence which has been bestowed on the anatomy of the ring. We fear there is too much truth in the following observation:

"I am well convinced, by many failures, as well as by what I have observed in my friends, that it is in vain, by *experiments on brutes*, to anticipate the years of practice and actual observation." P. 179.

The intestine may be in four different conditions. 1. In the sac, its function continuing, and the passage free. 2. In the sac, but adherent; its functions uninterrupted. 3. Retained by *incarceration;* that is, by its own distention, there being only stricture on the blood-vessels. 4. *Strangulation:*—The alimentary matter and blood obstructed—mortification impending. Mr. B. limits the subject, in the first place, to a consideration of the change from incarceration to strangulation; *i. e.* from obstruction of alimentary matter in the canal, to obstruction of the circulation and mortification of the gut.

The following Case is meant to elucidate the filling of he gut.

CASE 1. A woman was received for *Enteritis*. On examination, the pulse was quick and weak; features sharp and cadaverous; eyes wild; she moaned, and was in pain. Belly tumid; tender, especially near the groin; the pain described as rolling from side to side. A tumour in the groin, affected by the percussion of cough; appeared to have had stercoraceous vomitings for some time. It was plainly femoral hernia; but not in a state for reduction by pressure. In the operation, the vessels cut were inactive and did not bleed freely. The peritoneal sac was so weak, that, in pinching it up with the forceps, it burst, and dark coloured serum was discharged. The incarcerated portion of hernia was an inch and a half in diameter, and perfectly globular; it was of a dark red-brown colour, and adherent to the neck of the sac. Separated the adhesion by drawing up the neck of the sac; a little matter exuded; it was *fæculent!* Cut the sharp edge of the stricture; disengaged the gut; drew down the intestine gently. A dark sloughy spot appeared, but no opening. Pressure on the belly discharged liquid fæces from the sloughy spot. The incarcerated portion was now pressed, and emptied of clear gelatinous mucus. The torn part of the gut was brought down, so as to prevent the fæces escaping into the abdomen. She died.

*Observations.* The portion of intestine was probably pushed down during a strain. For a time the intestine lay empty, the stricture preventing the admission of alimentary matter. Circulation still went on in the vessels of the intestine. Secretion of mucus now took place, and distended the gut. Then there was imminent danger of strangulation. Finally, as the intestine filled, the angle of reflection over the sharp edge of the stricture increased [no, Mr. Bell; *decreased,* if you please*] every hour, till the coats became so tightly drawn against the edge of the stricture, that they were first gorged with stagnant blood; and finally the circulation was stopped. This is, we think, a good *rationale,* and we give Mr. Bell credit for it; but if he will use mathematical terms, he should know what these terms mean.

Mr. B. in this case shews the necessity of drawing down the intestine before the attempt at reduction; not only to bring the *firmer* part of the gut opposite to the stricture, but to examine the part. In hernia, the signs of danger,

---

* The angle at first must have been obtuse; and *decreasing,* became acute. Rev.

the hiccough, vomiting of fæces, filling of the belly, tormina, &c. have no direct relation to the strangulation of the gut included in the hernial sac. They proceed from the distention of the bowels within the belly, in consequence of obstruction; and do not accord with the actual danger, because *that* depends on the inflammation or mortification of the *incarcerated* portion.

Case 2. A gentleman, ætat. 63, perceived a small tumour in the groin, accompanied by pain and vomiting. He came thirty miles in a coach to town. Mr. B. found him animated and restless. He vomited easily, and without distress or retching. Mr. B. considered it hernia, and tried to reduce it; desisted; ordered a glyster: returned in three hours; became alarmed; put the patient in a hot bath, and tried the taxis again, but could not succeed. In a consultation, an eminent surgeon gave his opinion that it was not hernia! Next day, however, it was pronounced hernia; but the senior consultant saw no occasion for the operation. "*He was to leave town, and proposed to meet me the second day hereafter.* The patient died the succeeding day." From such "*Senior Consultants,*" good God, deliver us in the hour of danger!

Dissection. An inguinal hernia; and concealed by it, and under a load of fat, was found a small portion of intestine strangulated by the femoral ligament.

Case 3. A difference of opinion arose about an obscure tumour in the groin. Some said, bubo; some, hernia. The patient sided with the former; got up, and walked five miles. On dissection, the included intestine was found a putrid rag!

Case 4. Holden. Says his rupture is come down, and he cannot return it. A small soft tumour in the left groin, with a neck coming from under Poupart's ligament. Cannot be reduced, after bleeding, warm bath, &c. by the taxis. In the evening, Mr. Bell found him in great pain; no tension of the belly. An opening draught, some tinct. opii, and enema repeated. *Second day.* He asserts that the hernia is up; but it is not. He is alarmed, and won't tell a word of truth. Repet. ol. ricini. *Third day.* Has vomited. *Evening.* The tumour is harder. Has passed fæculent stools; vomited a chamber-pot full of fluid. Has hiccough.

Operation. Incision begun an inch and a half above Poupart's ligament, and continued below the tumour. The fat removed, the tumour appeared so distinct and round, that it did not seem to have any fascia covering it. Several successive layers were dissected off horizontally. The last layer was so strong and tense, that it was difficult to catch it with the forceps and pinch it up. On exposing the gut, a small portion of bloody serum escaped, and the gut rose freer from its bed. The colour of the intestine was that of a ripe cherry: no adhesions. The portion of intestine was so closely embraced, that the portion long pressed could not be drawn down and extricated; but the stricture being divided by the probe-pointed bistoury, the intestine was drawn down, and shewed a remarkable contrast with that previously down; it was pale. The intestine being gently pressed, the contents went up, and the flat piece of gut was easily reduced. No stitches were used; the integuments were brought together by adhesive straps, and retained by compress, roller, &c. He recovered without any bad symptoms.

Case 5. A woman, 30 years of age, had long been afflicted with reducible hernia. On Saturday it came suddenly down into her groin, and she could not get it up. The pain became intolerable, and continued night and day for a fortnight, during which time she had no evacuations. In the right groin there is a small tumour, compressible, and retentive of the impression of the finger; exquisitely painful; purgatives and glysters have been inoperative; stercoraceous vomiting for ten days. Mr. John Bell made a note at the time, "that he never saw a patient's death so apparently inevitable." She had the symptoms of internal gangrene; voice gone; cheeks hollow; visage ghastly; eyes turned up; eye-lids half open; arms motionless; hands cold; pulse thready. She was sunk and insensible; and only screamed deliriously when laid amidst the assistants.

Operation. A long incision was carried over the tumour, beginning pretty high up. The hernia appeared with its fascia; the latter divided, the hernia, somewhat flattened, sprung up. The sac and contents appeared, but required some dissection before it could be understood. Dissected all round, it seemed more like a tumour than a hernia, hanging pendulous by a narrow neck. The sac opened, omentum of a dingy straw colour presented, very hard and condensed by the pressure. This substance, drawn out and unfolded, seemed to occupy the sac so entirely, that the gut might have escaped notice. A small knuckle, or single turn of intestine, was found lurking behind the omentum; it was of a dark colour, and gorged with blood. The finger was pushed up to the neck of the sac; the bistoury introduced on the finger, when a nick of the knife was sufficient; the gut went up easily, and after it the omentum. Faintness, languor, vomiting, frequent low delirium, sunk eye, cadaverous countenance, followed, and recurred at intervals for three weeks; then came diarrhœa, which threatened to terminate the scene. From this state of abdominal inflammation she was saved by covering the abdomen with blisters, leeches, and by the warm bath. This was Mr. Bell's first public operation in the Edinburgh Infirmary. It does him great credit.

Case 6. A woman was received into the hospital, when a small femoral hernia was discovered. Her belly was much distended; vomited frequently; pain on pressing the belly, within the ligament. As she was said to have had evacuations downwards, the operation was postponed. *Consultation,* 10 *p. m.* Symptoms continue. Abdominal swelling greater; tumour not harder, but distinctly pinched; neck of the sac exceedingly narrow. Venæsection, warm bath, stimulating enemæ [enemata], and the taxis having been tried in vain, the operation was decided on.

During the operation, it was remarked that the fascia was so very thin as to resemble the peritoneal sac. The latter being pushed up with the forceps, burst like a bubble, and a quantity of clear serum burst out. The gut was very little distended, not at all discoloured, and adherent to the neck of the sac. The stricture on the neck of the sac was uncommonly narrow, and required great nicety to insinuate the directory. The probe-pointed bistoury being introduced on the groove, the stricture was cut, and the intestine easily reduced. The moment this was done, serum rushed from the abdomen, and the stream of fluid catching the edge of the sac, made a beautiful exhibition of it. On the 3d day, pain and swelling in the abdomen, but partial: yielded to leeches and blisters. She is now doing well.

Observations. Mr. B. considers it very dangerous to give drastic purges when the intestine is strangulated, as they overwork them when they have no means of unloading themselves. When we have ascertained that there is a hernia, that it produces vomiting, that it prevents evacuation downwards, that it cannot be reduced, every moment of delay is dangerous.

Case 7. A woman, 46 years of age, had a tumour in the site of femoral hernia. It was circumscribed, smooth elastic, and its neck felt going under Poupart's ligament. Abdomen near the neck of the sac tender on pressure. She had been bled; taken repeated doses of calomel; been put in the warm bath; had glysters thrown up; the taxis had been tried. She had now pain, in paroxysms of six minutes duration; vomited foul, turbid, fœtid fluid. The gentleman operated with great deliberation. With the blunt hook the several layers of membrane were raised up and dissected off; at length the sac assumed a transparent blackness. A gentleman present said, it was the intestine which arrested the operator's hand. He introduced the director under the ligament, and with the bistoury cut the stricture upwards and outwards. He attempted to reduce the hernia, but could not; cut the ligament a little more, and then reduced the whole tumour. She recovered rapidly.

Observations. The proper sac, in femoral hernia, is peculiarly thin and transparent: it is therefore, as in this instance, liable to be taken for gut and returned; but the practice is dangerous, as the stricture may continue, and the patient be lost.

Case 8. M. B. ætat. 50. In the right groin, an oblong tumour, extending from near the spine of the ilium to the labium. It is irregular, partly compressible, inflamed, painful to the touch. Abdomen tense and uneasy; pulse scarcely perceptible; countenance sunk. Has been subject to rupture twenty years. Present sufferings commenced twelve days ago, during which time she has been obstinately constipated. On making an incision, nearly five inches long, over the tumour, the parts were found adhering; and the cellular substance and fascia soft, and of a dirty yellow colour. The knife penetrated, at particular points, into a foul abscess, from which there was an oozing of matter. The sac and intestines were found entirely gangrenous, and broken into one mass, over which the fibres of the superficial fascia crossed like strings, and through which the fæces were forced out. The bistoury was now used, and the intestine freely opened. A small quantity of fæces was discharged; a little tepid water was thrown in, but did not return. The succeeding day, Mr. Bell's objections were over-ruled; the finger was introduced into the intestine, and the edge of the ligament divided by the bistoury. A considerable discharge of fæces followed; the patient was not relieved, but sunk and died.

Dissection. On opening the abdomen, much putrid air escaped. The whole of the intestines, particularly the small, very much inflamed; colour dark brown; ilium traced into a passage under Poupart's ligament; the two portions of intestine adhered together, and to the peritoneum; a little coagulated blood at the neck of the sac.

Observations. The intestine may be burst by ulceration on the sharp ligaments, or after mortification. When by the *latter*, the opening is to be enlarged; the surgeon is to introduce his finger, pass the point under the ligament, feel if the stricture be free to allow the fæces to pass; and if he can pass the tip of the little finger, no further operation is necessary. If the gut have come through a space so small as to impede the discharge, then the ligament is to be cut; not indeed by introducing the bistoury on the finger which is within the gut (for then the sound part of the gut is cut, and the abdominal cavity may be opened), but the parts must be dissected around the ring or neck of the sac; the edge of the ligament raised without injuring the intestine, and the firm or ligamentous parts only cut.

Mr. Bell observes, that he has generally found adhesions of the gut to the ring and mouth of the sac where there was mortification.

"This adhesion is not to be undone for the purpose of tying or sewing the sides of the gut together! Here experiments on brutes are good for nothing, and worse than nonsensical; they mislead."

He thinks the practice of passing a ligature through the mesentery, to retain the mortified gut from slipping into the abdomen, not much better. The best proceedure is this:—having cleared away the mortified portion of the intestine, attend carefully to the two canals which are left, the superior and inferior extremity.

"Pass a needle, carrying a seton cord, deep into the one orifice, and bring it out by the other. This will retain the intestine in its place, if that be necessary, and in due time it will form a communication between the two portions, and the continuity of the canal will be restored."

Mr. Bell's Sixth Report is wound up with observations on the amputation at the shoulder joint, on which we shall take the liberty of making a few animadversions. In this section of the work there evidently appears a tone of ill-will towards the brilliancy and eclat which have lately distinguished *military surgery*. Mr. Bell seems apprehensive that the "pack-saddle" and cock-pit shall eclipse the "theatre" and the hospital operation-room.

"Surgery has been improved in a remarkable manner, during the last fifty years, by the *lecturers* and hospital surgeons. Their efforts have been ably *seconded* in the practice of the army and navy. But every hospital surgeon and teacher claims the privilege of examining these operations, and the principles on which the military surgery has been conducted."

That is, if naval or military surgeons have done what the teachers were afraid to do, the teachers have now a right to find fault with these things; and though the operations were successful, if the principles (i. e. the theory) were contrary to the schools, they must now be censured and beaten down! Generous, liberal, disinterested teachers! And now for a specimen of our author's reasoning.

"The patients in military life are not drawn to their surgeons *by their reputation;* they have no power of selection; misfortune brings them under very absolute practitioners: *and this has often struck me as a reason why the army and navy surgeons should confine themselves to rules acknowledged by the general sense of the profession.*"

Pitiful effusion of crest-fallen jealousy!

"The compressing of the artery above the clavicle does *not* secure the patient against the loss of blood. It may be done, but is not to be depended on; for if it be, there will be *a great loss of blood.*"

Now we ask the profession, for candour is not to be expected from Mr. Bell on this point, whose opinion is to be taken in preference, the *teacher* who has amputated twice or thrice at the shoulder-joint, or the military surgeon who has done, and seen it done, one hundred times? Mr. Bell says, he "studied with all diligence under that excellent surgeon, Mr. Vance," at Haslar Hospital. Now we appeal to Mr. Vance, whether or not he considers pressure above the clavicle as safe. We know he does; for it is not many weeks since we saw him amputate at the shoulder-joint, without ever dreaming of *holding with the fingers and thumb the artery, while he cut it across below these.* Mr. V. boldly severed the limb, without any other precaution than supra-clavicular pressure, and not a drop of blood was lost. We have seen it performed *many times* in this way, and never saw hæmorrhage.

And now for the cloven foot. Speaking of the operation with pressure only, Mr. B. observes—

"I will admit this is very good practice, when a man is sitting on a *pack-saddle,* sublime amidst the Pyrenees, where a few jets of blood amidst the surrounding carnage is a matter of no moment: but, I believe, my friend will acknowledge, that in a London hospital it would make a very considerable sensation." 234.

In the first place, we deny the business of hæmorrhage *in toto*, from ocular evidence of its falsity. In the second place, we appeal to our readers, whether the above sarcasm be not a manifest proof of jealous or envious feeling. In the third place, we assert, that a British surgeon, "*sitting on a pack-saddle*," sublime amidst the Pyrenees, dressing the wounds of our brave countrymen who fought and bled at Talavera and Vittoria, is a nobler object than a self-important and self-appointed TEACHER, sublime amidst a score of gaping pupils, staring with amazement at the bones which he picked up in Haslar Hospital, or *taking in* the wonderful *tales* of Waterloo, collected during a four days' tour *after the battle was over!* *

---

### *Dr.* YOUNG, *on Consumptive Diseases.*

* "They would joke with me on my picking up the bones shattered by gun-shot, saying, these would make *fine stories* for lectures! *These bones were of much use to me.*" p. 228. Many true words have been spoken in jest! At all events, Mr. Bell has here, with much *naiveté*, cut a rod for his own back. We ask Mr. Bell, whether a single operation of *his* succeeded at Brussels?

